# Differentiating Categories of Violent Adolescent Offending and the Associated Risks in Police and Youth Offending Service Records

**DOI:** 10.1177/0306624X211058960

**Published:** 2021-12-03

**Authors:** Sally-Ann Ashton, Michael Valentine, Bonnie Chan

**Affiliations:** 1 Edge Hill University, Ormskirk, UK; 2 Merseyside Police, Liverpool, UK

**Keywords:** adolescent offending, violent offending, adverse childhood experiences, police records, youth offending supervision

## Abstract

Historical risk assessment forms for a sample of 173 males with a history of violent offending and under supervision by Merseyside Youth Offending Services (YOS) were investigated. Subsequent arrest records were scrutinised in order to obtain a better understanding of the relationship of social and psychological risk factors to offending behavior. The mean age of the sample at the point of contact with YOS was 16.01 (*SD* = 1.37) with a range between 12 and 18 years. Assault was associated with solo expressive offending, a history of domestic violence, low school attendance and an inability to control impulsivity and aggression. Robbery was associated instrumental and escalated violent offending, psychological disorders, and deviant groups, including family criminal involvement. Risk assessments by professionals and the young people indicated that substance misuse co-occurred with robbery. The findings suggest that solo offenders commit the majority of violent offences and that targeted interventions should distinguish between expressive and instrumental offending.

## Background

### Adolescent Violent Offending

Aggression is a fundamental component of violent offending but can be divided into different categories of hostile/expressive and instrumental, depending on the goals of the perpetrator (Anderson & Bushman, 2002; Feshbach, 1964). Researchers make a further distinction between expressive aggression as “reactive” and “impulsive” versus instrumental aggression as “proactive” and “premeditated” (Anderson & Huesmann, 2007). However, as Allen and Anderson (2017) emphasize, acts of aggression do not always fall within a single type or hostile or instrumental and suggest a “dimensional” rather than “dichotomous” approach to categorisation (Anderson & Carnagey, 2004). Central to this framework is the degree to which the behavior was planned, and whether the consequences considered; the extent to which the goal of the offence was benefiting the perpetrator compared to harming the victim; and the degree to which the perpetrator was hostile or agitated during the commissioning of the offence (Allen & Anderson, 2017).

Aggression and violence can also be explained by the need of an individual to self-preserve or self-promote in interpersonal relationships (Toch, 1969). It has been suggested that most violent episodes stem from a learned strategy by perpetrators to deal with previous interpersonal relationships, and in order to maintain status in an aggressive subculture (Salfati, 2000; Toch, 1969). Gangs are an example of such a subculture, and gang violence can be both instrumental and expressive (Cuevas & Rennison, 2016). However, the lack of homogeneity in structure and experiences of UK gang members has led some to question the effectiveness of the label (Bennett, 2004; Curry, 2015).

Research has demonstrated that there is a relationship between violent crime and co-offending irrespective of gang status and that temporary co-offending groups present a heightened risk for certain categories of crime (Warr, 2002). Co-offending is associated with affray, burglary, robbery, vehicle taking, arson without the intention of endangering life, and drug use (Hodgson, 2007). Adolescents and young adults are more likely to commit violent crime when in the company of others; explanations for this include the depersonalization of violence mitigating the responsibility of individuals in a group (Alarid et al., 2009). Also for consideration is also the impact of a more experienced or aggressive offender within a group escalating violence during the commissioning of either a planned or opportunistic offence (Conway & McCord, 2002).

However, not all violent offences are equally associated with groups. A study by the US Department of Justice (USDJ; Oudekerk & Morgan, 2016) found that simple assaults were the most common form of violent victimizations for those who acted alone, and that more non-fatal violent acts were committed by solo offenders. In contrast, more than half of the serious violent crimes, including robbery, rape, and aggravated assault, were committed by groups of adolescents, supporting prior research on the escalation of violence in groups (Conway & McCord, 2002). The USDJ research also found that adolescent and young adult offenders were more likely to use a weapon when engaging in serious violent acts alone (Oudekerk & Morgan, 2016).

Flexibility has been found among long-term prolific offenders who are prepared to act both with others and alone (McCord & Conway, 1996; Reiss, 1988; Reiss & Farrington, 1991). Research on longitudinal data from the US has, however, demonstrated that persistent and prolific offenders vary their offending style much earlier in their criminal careers (Ashton et al., 2020; Goldweber et al., 2011). During adolescence such individuals have the potential to influence peers and to instigate crimes; recruiters have also been found to belong to a category of life-course persistent offenders and to present a greater risk than those who limit their style to either solo or co-offending (Moffitt, 1993).

Those who fulfil principal roles in co-offending groups are typically older, and are often family members (Reiss, 1988; van Mastrigt & Farrington, 2009). Furthermore, co-offending with more experienced individuals has been found to extend future offending trajectories for certain categories of offence, such as burglary (Hodgson & Costello, 2006). This phenomenon is not uncommon. A prison-based study showed that 40% of prisoners could identify a male “mentor” who encouraged them to become involved with crime (Morselli et al., 2006). Child criminal exploitation (CCE) is recognized as part of the expansion of illegal drug markets in the UK (Densley et al., 2020). Studies have consistently shown that young people who are involved with adult drug dealers and crime groups suffer from physical and emotional abuse (Ashton & Bussu, 2020; Moyle, 2019).

Weapon carrying and use can also be expressive or instrumental (Sheley & Wright, 1993). It has been suggested that young males carry knives for different reasons, including a perceived need for protection, fear of crime, and to enhance their status (Riggs & Palasinski, 2011). Gang membership and drug selling can also increase an individual’s perception of the need to carry a knife for protection (Riggs & Palasinski, 2011). However, a study in Edinburgh showed that the risk factors associated with knife crime and gang membership were different. Knife carrying was associated with self-harm, social isolation, low self-esteem and lack parental support (McVie, 2010). In contrast, young people who were gang involved were associated with social disadvantage and high crime areas. More generally, knife carrying has been associated with peer antisocial influence and instrumental offending and protection (Dijkstra et al., 2012).

### Assessment Risk Factors Associated with Adolescent Violent Offending

The AssetPlus is an assessment is the standard Youth Justice Board assesment tool for Youth Offending Services in England and Wales (Hampson, 2018). The assessment seeks to identify risk and protective factors, under the headings of personal, family and social. Thus enabling practioners to focus work and interventions on life goals, desistance, protecting others, keeping safe and repairing harm (Hampson, 2018; Youth Justice Board, 2014). The forms contain a combination of professional and reflective self-assessment and draw upon client questionnaires and official data to build an assessment. Identifiable risks include: exposure to violence (familial and antisocial peers with a focus on gang membership); substance and alcohol use; family relationships; childhood trauma; mental wellbeing; impulse control; and child criminal and sexual exploitation. These factors also relate to the young person’s routine activities and relationship to education or work.

Exposure to community violence and violent victimization are associated with an increase in aggressive offending (Baskin & Sommers, 2014). Gang members are twice as likely as non-gang members to be both perpetrators and victims of crimes (Pyrooz et al., 2014). This risk is significantly reduced as soon as an individual leaves the gang (Ashton et al., 2020a), but this does not necessarily moderate the longer term impact of violent trauma. Furthermore, increased exposure to violence is associated with those who mix their style of offending during late adolescence, suggesting that instigators of group offending present a higher risk, irrespective of gang membership (Ashton et al., 2020b).

Some researchers have suggested that peer influence is greater for those who begin offending during their adolescence, because their reasons for committing crimes can be socially motivated and relate to status (Weerman, 2003). It is important, when considering the effect of delinquent peers, to distinguish between persistent and age-specific offenders, motivation for offending, and category of offence (McGloin & Povitsky Stickle, 2011). For example, peer delinquency is more strongly associated with non-violent, income generating offences (Kalvin & Bierman, 2017). Theories that associate low self-control with offending (Gottfredson & Hirschi, 1990) are also relevant to an individual’s ability to resist the influence of delinquent peers (McGloin & O’Neill Shermer, 2009; Wright et al., 2001).

Individuals with low self-control are less likely to consider the consequences of their actions. Low impulse control has been associated with increased group offending (Hirschi & Gottfredson, 2000; McGloin et al., 2008), and it has been suggested that individuals with poor self-control may be drawn to others who share the same deficit (McGloin & O’Neill Shermer, 2009). Studies utilizing longitudinal data from the Pathways to Desistance Project have demonstrated the association between adolescent group offending and lower levels of impulse control (Ashton et al., 2020b; Goldweber et al., 2011). Using trajectory analysis on the same data, other researchers found that less mature individuals are likely to be persistent and offend more frequently (Steinberg et al., 2015). Psychosocial maturation is a dynamic risk factor for adolescents; and its increase has been associated with desistance from crime for adolescent-limited offenders (Moffitt, 1993). Research suggests that psychosocial maturity continues to develop into the mid-twenties and explains offending desistance in early adulthood (Ashton & Ioannou, 2020; Monahan et al., 2013).

Understanding the role of the family in risk assessments of offending behaviors is also crucial for the implementation of successful interventions (Marcus, 2017). A study of young people who had been charged with domestic violence offences found that adolescents who used violence against their parents had been exposed to physical or sexual abuse at home, or witnessed intimate partner violence (Routt & Anderson, 2011). Researchers also confirmed known indicators of family violence; half of their sample demonstrated behavioral problems at school and had low attendance rates; 39% their sample presented with mental disorders; and 22% had a substance abuse problem. Perpetration of domestic violence can be learned behavior; however, studies have also shown an association between domestic violence within a family and poor impulse control, impeded cognitive processing, and low self-esteem for children and adolescents (Routt & Anderson, 2011). Domestic violence can also impact on offending behaviors; young male offenders who have been physically abused have been found to commit higher levels of violent, property and felony offences (Herrera & McCloskey, 2001).

Three components associate illegal drug use with violence: psychopharmacological effects of different drugs, the need to finance a drug habit, and the occurrence of violent behaviors that are linked to the drug market (Goldstein, 1985). This last component formed the focus of the British Government’s violence reduction strategies; specifically, the involvement with organized crime and street gangs in the sale of drugs (Home Office, 2016, 2018). The relationship between substance abuse and serious violence is complex, because the two behaviors have many of the same risk factors (Hawkins et al., 1992; Home Office, 2018). However, long term effects of the use of cannabis can cause paranoia in adolescents (Johnson et al., 2000).

A study of juvenile offenders indicated that 65% demonstrated comorbidity for mental health and substance misuse disorders (Hussey et al., 2007). Although it is widely accepted that anxiety disorders such as PTSD and ADHD have a strong relationship with aggressive behavior, depressive disorders are also prevalent among adolescent offenders (Grisso, 2008).

## The Present Study

The aims of the study are: To explore offending patterns in a historical sample of young people who had committed at least one violent offence and who were under supervision of Youth Offending Services. To investigate which risk factors on the Youth Offending Service AssetPlus evaluations are associated with expressive and instrumental violent offending. To consider how to identify high risk individuals and the most appropriate interventions.

## Method

### Sample

Two Youth Offending Services (YOS) selected historical AssetPlus forms for their local authority with a view to understanding more about criminal exploitation and violent offending. To be included in the study, participants had to have committed at least one of the following offences: Assault, carrying a knife/weapon, public order section 4, criminal damage, robbery, intent to supply class a drugs. The sample consisted of 172 males from two local authorities in Merseyside.

### Materials

Asset Plus forms consist of a core record: Personal information; parent/carer details; current and previous antisocial behavior; risk of harm to the young person; record of contact with other services; details of the young person’s circumstances; and a summary of key actions from the intervention plan. Information gathering: Personal family and social factors; offending and antisocial behavior; foundations for change; and self-assessment. Explanations and conclusions: Explaining offending behavior patterns; considering potential future behavior; reoffending rating and risk of serious harm rating (Youth Justice Board, 2014).

### Procedure

Data from the Asset Plus forms was coded into categorical variables of present or not present. Arrest data was then checked on the regional police computer, and charges were checked on the Police National Computer in order to check out of area. All data was recorded and stored on a secure drive in the Department of Performance, Analytics and Evaluation at Merseyside Police Headquarters. As soon as data had been cross checked it was anonymised and transferred from spreadsheets to SPSS for analysis.

### Analysis

Descriptive statistics were obtained for sample demographics, offending frequency, style and variety. A two-way analysis of variance (ANOVA) investigated the relationship between offending style and number of arrests. An independent sample *t*-test was undertaken to compare the number of arrests for those who had been arrested with an adult compared to those who had been arrested with peers at any point in their offending timeline. Data from the Asset Plus forms and regional police computer was analyzed using Smallest Space Analysis (SSA-I; Lingoes, 1973), an established multi-dimensional scaling procedure that shows the relationship of every variable to every other variable. Each variable is plotted as a spatial representation showing the associated correlations between them. The following relationships were investigated: Offence arrests; violent offences and professional risk assessment; and violent offences and self-assessed risk. Pearson product-moment correlation was undertaken for the scale data of the self-assessed risk and assault and to investigate the relationship between LSOA rating and number of arrests; and a Chi-squared test was used to investigate the relationship between knife carrying and other offences.

## Results

### Sample Demographics

At the age of contact with YOS the mean age was 16.01 (*SD* = 1.37) with a range between 12 and 18 years. The mean age in January 2020 when the arrest data were checked was 17.27 (*SD* = 1.43, range 13–20 years). 80.1% of the sample identified as White British; 7% (*n* = 12) as Mixed “other background”; 5.9% (*n* = 10) as White “other”; 1.8% (*n* = 3) as Asian/White mixed; 2.9% (*n* = 5) as Black “other”; and 0.6% (*n* = 1) as: Black British Caribbean, Mixed Black Caribbean/White; Mixed Black African/White; Mixed “any other background.” The Lower Layer Super Output Areas (LSOA) rating was heavily skewed with the majority of the sample living in areas of higher deprivation. The mean LSOA rating was 5749.96 (*SD* = 7746.10).

YOS records indicated that 22.4% (*n* = 37) of the males had been in foster care or a care home at some point in their lives. Child Services had been involved with 58.2% (*n* = 95) of the sample; 18.6% (*n* = 31) was recorded as having been psychologically or physically abused at home. The Asset Plus forms reported that 27.1% (*n* = 48) had witnessed domestic violence at home; however, the regional police database listed 55.6% (*n* = 95) of the sample had been present at the time of a police call out for domestic violence, showing a considerable discrepancy between the police and youth offending reporting. Family involvement in crime was reported as 30.9% (*n* = 50).

The most commonly reported offence was assault, with 69% of the sample arrested for this crime; the majority committed this offence alone. Criminal damage, Section 4 public order offences, and knife carrying followed similar patterns. The most commonly reported income generating offence was theft, followed by burglary. The higher number of adult offenders who were reported at the time of arrest for burglary is indicative of criminal training.

The mean number of arrests reported on the police regional database was 10.48 (*SD* = 10.56) with a range between 1 and 57 arrests, showing a high degree of variation in the offending profiles. A one way between groups analysis of variance (ANOVA) was conducted to investigate the impact of offending style on offending frequency. The sample was divided into three groups according to their overall offending style of solo (only alone), co (only with other people), and mixed (those who were arrested both alone and with other people). There was a statistically significant difference all groups Welch’s *F*(2, 88.49) = 65.43, *p* < .000. The effect size, calculated using eta squared, was .13 (medium). Post hoc comparisons using the Games-Howell test, in recognition of the unequal sample sizes and variance, indicated that the mean score for mixed style offenders *n* = 114 (*M* = 13.07, *SD* = 11.47) was significantly higher than those who only offended alone *n* = 44 (*M* = 5.41, *SD* = 4.79), and those who only offended in the presence of other people *n* = 7 (*M* = 1.57, *SD* = 0.54). The mean scores for those who were only arrested alone was also significantly higher than those who only co-offended. The number of arrests for those who had been arrested with an older person present (*n* = 97; *M* = 13.57, *SD* = 12.24) was significantly higher than who offended alone or with peers (*n* = 68; *M* = 6.22, *SD* = 5.12), *t*(146) = 2.01, *p* = .05, and the effect size, calculated using Hedges’ *g*, was medium.

### The Relationship Between Arrest Offences

**Figure 1.**
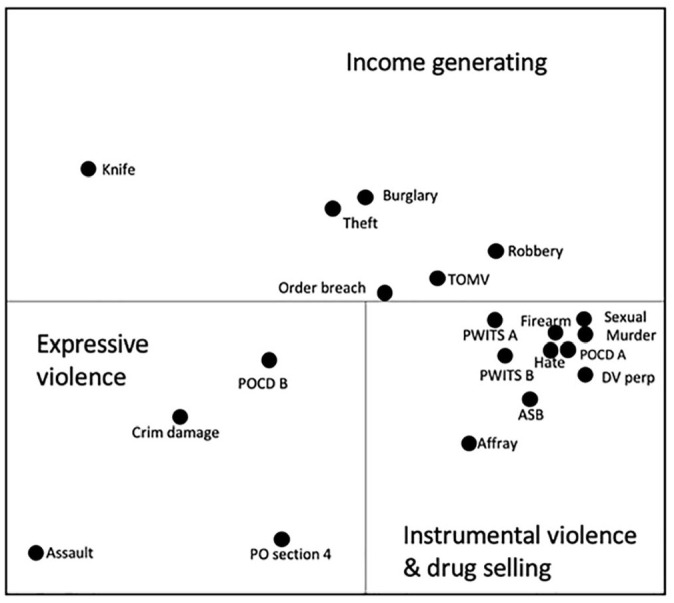
Three-dimensional smallest space analysis of arrest records with regional interpretation. *Note. Coefficient of alienation* = .10. PWIT = possession with intent to supply class A or B drugs; DV perp = young person is a perpetrator of domestic violence; ASB = Antisocial behaviour; PO section 4 = threatening or abusive behavior; POCD = possession of a controlled drug class A or B.

A smallest space analysis (Figure 1) was performed to investigate the relationship between arrest offences. The category of offence was coded as present or not present for each of the participants. Two main regions emerged on the SSA plot. At the top are mainly income generating offences, along with breach of an order or bail condition. The lower region of plot divides into two separate areas. Clustered together are more serious drug related offences, including intent to supply class A drugs, possession of a firearm, and violent offences associated with aggression and lack of impulse control. To the lower left region is assault, criminal damage, and section 4 offences, which are associated with possession of cannabis. Drug possession and sales are therefore associated with violent behaviors. Carrying a knife is positioned away from the main variable groups; the space around it suggesting behaviors that were not reported. There is a clear escalation of violent offending in the lower section of the plot. This might suggest adult involvement with increased involvement in drug selling and taking.

Since the relationship between knife carrying and other offences was not clear from the SSA, a chi-squared test of independence was also performed to examine the relationship between carrying a knife and other offences. Significant relationships were found with: burglary *χ*^2^ (1, *N* = 172) = 9.91, *p* = .002; theft of a motor vehicle *χ*^2^ (1, *N* = 172) = 8.53, *p* = .003; section 4 public order offences *χ*^2^ ( 1, *N* = 172) = 4.54, *p* = .03; and possession of a controlled class B drug *χ*^2^ (1, *N* = 172) = 4.48, *p* = .03. The strongest relationship being with burglary and theft of a motor vehicle.

It is important to note that no significant correlation was found between LSOA rating and number of arrests; individuals from more socially deprived areas were not arrested significantly more offences. The mean LSOA rating for the male sample was 5750 (*SD* = 7746). The mean for those who had at least one arrest for assault was higher at 6040 (*SD* = 7901); and for robbery the mean was lower at 4,519 (*SD* = 5,329).

### Professional Risk Assessment and Violent Offending

**Figure 2.**
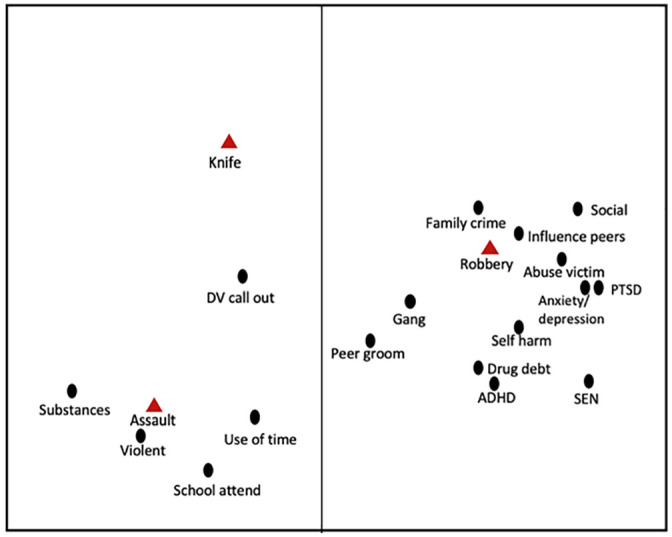
Three-dimensional smallest space analysis of professional risk assessment and violent offences with regional interpretation. *Note.* Coefficient of alienation = .09

A smallest space analysis (Figure 2) investigated the relationship between robbery, knife carrying and assault to professional risk factors. Assault was associated with a lack of routine activities, drugs, and violent behavior. At the time of the professional assessment not all of the young people would have demonstrated a pattern of violent offending, suggesting that assessment can identified risk early. Domestic violence calls outs from the police database were also associated with assault; the relationship may represent learned behavior or childhood trauma. Knife carrying was situated in this half of the plot but appears to be independent, suggesting that there are missing risk factors associated with this particular offence. It is possible that young people carry knives for different reasons; this would cause the variable to be positioned away from the main clusters of risk.

Robbery was associated with mental health disorders and illnesses, the social risks of gang involvement, peer grooming and family involvement with crime. Inappropriate social behavior and drug debt were also associated with this violent, income generating crime.

### Self-assessed Risk and Violent Offending

**Figure 3.**
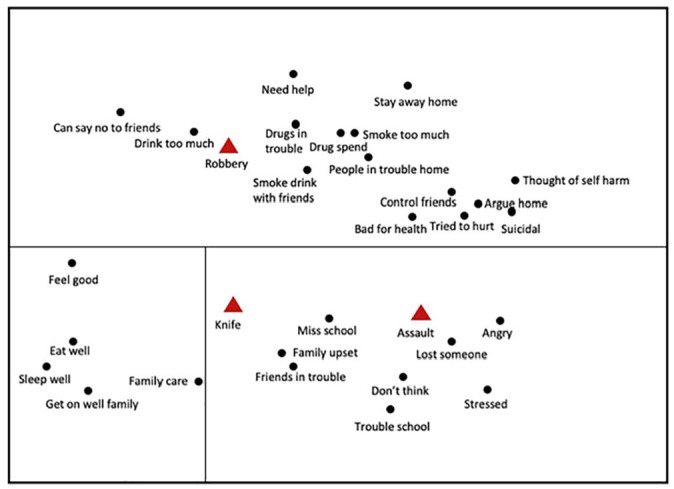
Three-dimensional smallest space analysis of professional risk assessment and violent offences with regional interpretation. *Note.* Coefficient of alienation = .19

A smallest space analysis (Figure 3) investigated the relationship between robbery, knife carrying and assault to self-reported risk factors. Protective risk factors were positioned together in the same region of the plot. Assault and carrying a knife were associated with anger/stress and not thinking about consequences. This could suggest aggression and lack impulse control, a dynamic developmental risk factor. Trouble at school can be explained by the aggressive behavior and exclusion. The family being upset is potentially a protective risk factor, although this could equally demonstrate tensions in the home. Once again, knife carrying is in the same region as assault but is positioned away from the main cluster.

Robbery was associated with substance misuse, problems at home and staying away from home. As a risk, staying away from home is generally associated with adult grooming and county lines. However, a degree of perceived control by the young person is present in saying no to friends. The presence of the ability to control peers in this region of the plot could suggest the transfer of skills and control that were acquired through adult or peer instruction to others. The co-existence of substance and alcohol misuse and mental illness suggest that a comprehensive professional approach is required to support young people who commit this crime.

## Discussion

### Offending Patterns

**Figure 4.**
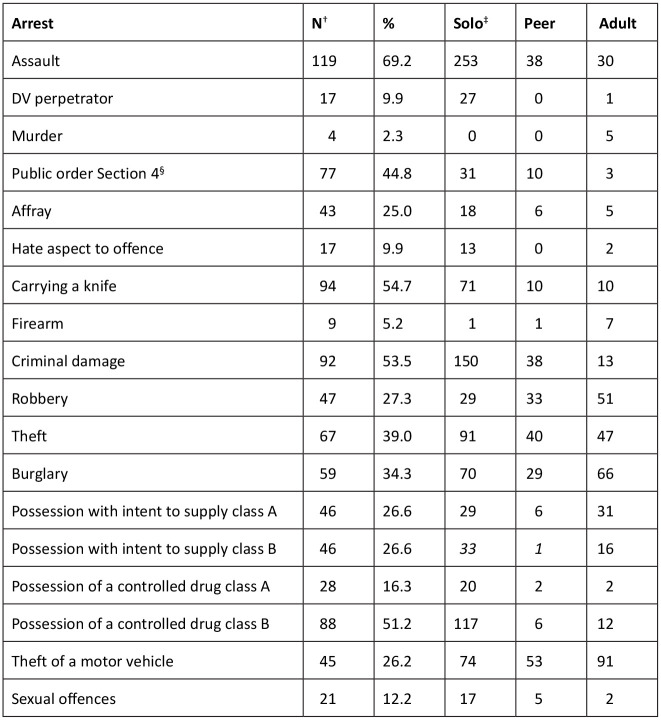
Arrests and offending style. *Note.* ^†^Number of participants reporting at least one arrest in the category. ^‡^Overall number of offences reported. ^§^Causing fear or provocation of violence, including racially/religiously aggravated.

Assault by an individual was the most commonly recorded arrest (Figure 4). Solo offenders were also responsible for the majority of other violent offences, including the carrying of knives. This finding accords with USDJ research (Oudekerk & Morgan, 2016) and suggests that weapon carrying may be a substitute for the protection that an offending group affords to an individual (Riggs & Palasinski, 2011). Knife carrying appeared in the same region as acquisitive crimes suggesting an instrumental relationship; this was confirmed by the significant correlation with burglary and theft of a motor vehicle (Sheley & Wright, 1993).

Solo offenders had a significantly higher number of arrests than those who offended only in the presence of others, indicating that autonomy is associated with increased offending. As with prior research recognizing the category of mixed-style offender, this group had a significantly higher number of arrests than both solo and co-offenders (Ashton et al., 2020b). Since offending style is more easily determined than gang membership status, authorities could utilize this behavior as a high-risk marker.

Another significant indicator for arrests was the presence of an older offender anywhere on the offending timeline of the young people. This suggests that the focus of criminal grooming and exploitation needs to be extended beyond drug trafficking (Morselli et al., 2006). Group offending was also associated with a move from expressive to instrumental offending with an escalation of violence, supporting prior research (Alarid et al., 2009; Conway & McCord, 2002; Oudekerk & Morgan, 2016).

Acquisitive crimes by deceit or force were positioned in the upper region of the SSA plot (Figure 1). The high number of arrests for burglary was suggestive of adult involvement if not during the arrest offence then in training (Hodgson & Costello, 2006). It is also possible that the solo burglaries involved an adult, who wasn’t present at the time of arrest but who was involved with the offence planning and orchestration. Interventions therefore need to focus on the risk factors associated with individuals and look more broadly at criminal exploitation; this is currently identified for drug selling but does not appear to include other crimes. This is probably because the focus for adult grooming is drug selling (Densley et al., 2020); however, the range of crimes involving child exploitation is more varied.

Three stages of violence were identified in Figure 1: expressive violence including assault; instrumental income generating violence including robbery; and an escalation of seriousness to instrumental and expressive violence, including sexual offences, firearms offences, homicide, and possession with intent to supply drugs; all of which suggest involvement in organized serious crime. These behaviors were also associated with antisocial behavior, affray, and domestic violence, suggesting a lack of control. These findings support the need for a dimensional approach to understanding violent offending, as prescribed by Allen and Anderson (2017).

### Professional Risk Assessment and Violent Offending

Instrumental and expressive violence were positioned in distinct regions of the SSA plot (Figure 2). In accord with prior research assault was associated with a lack of routine activities, substance misuse, a history of violent behavior in the home, and police call outs for domestic violence (Routt & Anderson, 2011). This also explains why assault is associated with lone offending and suggests a lack of control rather than planning. Robbery on the other hand was associated with social criminogenic risks including delinquent group membership, family crime involvement and peer grooming, supporting prior research in the US (Oudekerk & Morgan, 2016). The vulnerability of individuals who had been arrested for these offences is also evident, with victimization, mental disorders and special educational needs appearing in the same region of the plot (Grisso, 2008). The ability to influence peers was also located in the same region, suggesting that the young person may present a risk to others in their social networks or engage in mixed style offending. It is also possible that it reflects the process of criminal skill development. These findings give context to the data for robbery arrests; the majority were in the presence of an adult and/or peer and may explain why other studies have also found robbery to be associated with group offending (Conway & McCord, 2002; Oudekerk & Morgan, 2016). Previous research had suggested that peer delinquency is more strongly associated with non-violent acquisitive offending (Bernburg & Thorlindsson, 1999; Kalvin & Bierman, 2017). The presence of the risk of drug debt in the same region may explain part of the motivation for this acquisitive crime, and also supports prior research on the use of drug debt to coerce young people into drug selling and trafficking (Ashton & Bussu, 2020; Moyle, 2019; Storrod & Densley, 2017).

### Self-assessed Risk and Violent Offending

The self-assessment results echoed those of the professional risk evaluations. Assault and knife carrying, which were predominantly solo offences, were associated with anger, stress and not thinking about consequences. Previously, low impulse control has been connected with group offending (Hirschi & Gottfredson, 2000; McGloin et al., 2008). However, the Merseyside data suggests that expressive solo offending transitions to instrumental violent offending when individuals become involved with deviant groups (Figure 4). In contrast, robbery was associated with problems at home, substance misuse and self-harm; suggesting the need for programs that involve the family and also address substance use and mental illness (Marcus, 2017). Again, involvement with adult criminal groups was associated with the ability to control and influence peers, reflecting the cycle of criminal exploitation. Protective factors were clustered in a distinct region but were close to the family being upset with the young person’s behavior and expressive violence.

## Conclusion

In concurrence with prior research in the US (Oudekerk & Morgan, 2016), two profiles of violent offender emerged from the study. Solo offenders committing expressive crimes and group offenders who associated an escalation of instrumental violence. Knife carrying was associated with solo arrests. Mixed-style offending and the presence of an adult co-offender on an individual’s offending timeline were associated with significantly more arrests. Both professional and self-assessed risk demonstrated that different variables were associated with assault and robbery. Professional risk assessment showed that robbery was associated with psychological disorders, peer delinquency, gangs, controlling peers and family criminal involvement. Assault was associated with prior domestic violence, problems at school, inappropriate use of time, and anger. The only key difference between the two forms of assessment was the role of substance misuse; this was associated with assault by professionals and robbery in the self-assessments. The findings suggest that targeted interventions are necessary for the two categories of violent offending.
